# Scavenging by threatened turtles regulates freshwater ecosystem health during fish kills

**DOI:** 10.1038/s41598-020-71544-3

**Published:** 2020-09-17

**Authors:** Claudia Santori, Ricky-John Spencer, Michael B. Thompson, Camilla M. Whittington, Thomas H. Burd, Samantha B. Currie, Timothy J. Finter, James U. Van Dyke

**Affiliations:** 1 School of Life and Environmental Sciences, The University of Sydney, Camperdown, NSW 2006 Australia; 2 School of Science, Hawkesbury Institute for the Environment, Western Sydney University, Richmond, NSW 2753 Australia; 3 School of Molecular Sciences, La Trobe University, Wodonga, VIC 3689 Australia

**Keywords:** Ecology, Ecosystem ecology, Ecosystem services, Freshwater ecology

## Abstract

Humans are increasing the frequency of fish kills by degrading freshwater ecosystems. Simultaneously, scavengers like freshwater turtles are declining globally, including in the Australian Murray–Darling Basin. Reduced scavenging may cause water quality problems impacting both ecosystems and humans. We used field and mesocosm experiments to test whether scavenging by turtles regulates water quality during simulated fish kills. In the field, we found that turtles were important scavengers of fish carrion. In mesocosms, turtles rapidly consumed carrion, and water quality in mesocosms with turtles returned to pre-fish kill levels faster than in turtle-free controls. Our experiments have important ecological implications, as they suggest that turtles are critical scavengers that regulate water quality in freshwater ecosystems. Recovery of turtle populations may be necessary to avoid the worsening of ecosystem health, particularly after fish kills, which would have devastating consequences for many freshwater species.

## Introduction

Freshwater ecosystems support ~ 9.5% of all species^1^, but they are increasingly impacted by human activities such as agriculture, pollution, and overexploitation^2^. These activities damage freshwater habitats, degrade water quality, and threaten biodiversity^2^. Mass deaths of fish (‘fish kills’) are often natural events, but human impacts are increasing their frequency and severity^3^. Fish kills degrade water quality by increasing the concentrations of ammonia and nitrates, and driving phytoplankton and cyanobacteria blooms, which decrease dissolved oxygen concentrations^4,5^. Together, these effects can lead to devastating biodiversity losses and ecosystem breakdown^6,7^. Fish kills also have human impacts; bacterial and cyanobacterial blooms can cause serious illnesses, such as botulism^8^.

Recently, possibly over a million fish died in three massive fish kill events in the Darling River, Australia^9^, events that were linked to over-extraction of water and excess agricultural runoff^10^. In the same system, the Australian Government plans to target invasive common carp (*Cyprinus carpio*) with cyprinid herpesvirus-3 (CyHV-3^11^). Viral control could leave millions of tonnes of carp carcasses rotting in river systems that supply drinking water and agricultural irrigation^12^, representing a major risk for humans^13^.

Aquatic scavengers play a key ecological role during fish kills by consuming carcasses, and possibly reducing the harmful effects of carrion decomposition on water quality^14,15^. Turtles are important scavengers in freshwater systems, but their global declines mean that this role is being lost^16^. In the 1980s, India recognised this issue and reintroduced about 40,000 turtles into the Ganges River to address its pollution problem of decomposing human remains^17^. In the Murray–Darling Basin of south-eastern Australia, in the 1970–1980s turtles were estimated to eat up to 430 tonnes of carrion per day^14^, but all three endemic species are currently threatened^18,19^. Since 1976, in parts of their range *Chelodina longicollis* has declined by > 90%, and *Emydura macquarii* by 69%^18^.

Here, we tested the hypotheses that scavenging by freshwater turtles (1) accelerates carrion decomposition, and (2) regulates water quality during a fish kill. First, we used a field experiment to test whether turtles are important scavengers. We tested this hypothesis by experimentally excluding turtles from carp carcasses, and comparing decomposition rates of carcasses that turtles could not access to ones that turtles could access. Then, we used a mesocosm experiment to determine whether turtle scavenging affects water quality during a fish kill. We expected to find evidence that turtles are important scavengers in the Murray–Darling Basin, and that water quality is tightly linked with the rapid removal of rotting carrion.

## Results

### Field experiment

**Figure 1:**
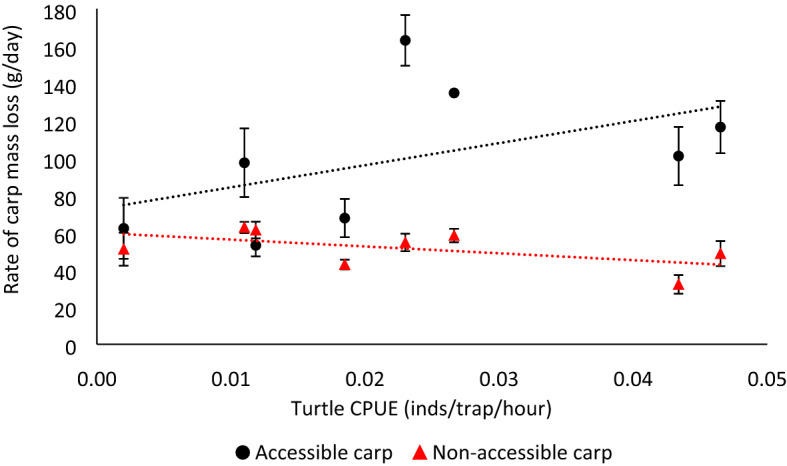
Carp mass loss rate (mean ± SE) increased with increasing turtle CPUE when carcasses were accessible to turtles, but not when carp were not accessible. The slope of the linear regression between turtle CPUE and rate of mass loss of accessible carp (black dots) was 1,186.98 ± 868.46, whilst the slope of the non-accessible carp (red triangles) was -367.89 ± 221.90. *Inds* individuals.

The total catch-per-unit-effort (CPUE) of the two sites directly open to the river was higher than in the two sites not directly connected to it (Supplementary Table S1). *Emydura macquarii* was the most abundant species captured. We observed shrimps (*Paratya australiensis*), prawns (*Macrobrachium australiense*) and crayfish (*Cherax destructor*) in both accessible and non-accessible carp boxes. A medium-sized *E. macquarii* was observed eating an accessible carp. No other potential scavenger such as birds or rakali (*Hydromys chrysogaster*) was observed in the proximity of the carp carcass deployment sites or approaching the carcasses. Carp accessible to turtles lost mass more rapidly than those not accessible to turtles (Supplementary Fig. S1), and the rate of mass loss increased with increasing turtle CPUE (*F*_1,69_ = 24.1, *p* < 0.001; Fig. 1; Table 1).

**Table 1:** Type 3 analysis of variance table calculated with Satterthwaite’s method^20^, for the linear mixed-effects model, with the rate of carp mass loss as a dependent variable.

	Num DF	Den DF	F-value	*p* value
CPUE	1	70.9	0.317	0.575
Carp access (yes/no)	1	69.3	1.708	0.195
Carp initial mass (g)	1	69.1	4.035	0.049
CPUE * Carp access (yes/no)	1	68.9	24.14	< 0.001
CPUE * Carp initial mass (g)	1	69.7	0.312	0.587
Carp access * Carp initial mass (g)	1	69.3	1.752	0.190

### Mesocosms experiment

**Figure 2:**
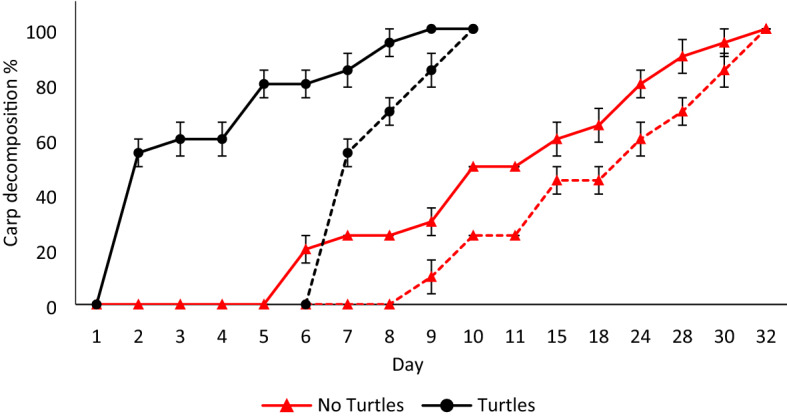
Decomposition percentage (mean ± SE) of the two carp carcasses (solid line first carp, dashed line second carp), in mesocosms with (black lines, circles) or without (red lines, triangles) turtles. See Supplementary Table S10 for a full description of decomposition estimation methods.

In mesocosms with turtles, carp carcasses were completely removed three times faster compared to control mesocosms (*F*_1,8_ = 482.65, *p* < 0.001; Fig. 2; Supplementary Table S2). When turtles were absent, time to total decomposition was 27.4 days (± 1.05 SE) for the first carp, and 25.2 days (± 0.39 SE) for the second carp. When turtles were present, time to total decomposition was 6.4 days (± 1.05 SE) for the first carp, and 3.6 days (± 0.39 SE) for the second carp.

Principal component (PC) 1 and PC2 had eigenvalues greater than one, and therefore were used for further analysis (Supplementary Table S3). There were no significant four- or three-way interactions between the explanatory variables of the GLMM for PC1 or PC2 (Supplementary Tables S4, S5), therefore we also computed simplified models. Turtle presence significantly affected both PC1 and PC2, and was dependent on day number for PC1 (Supplementary Tables S6, S7).

**Figure 3:**
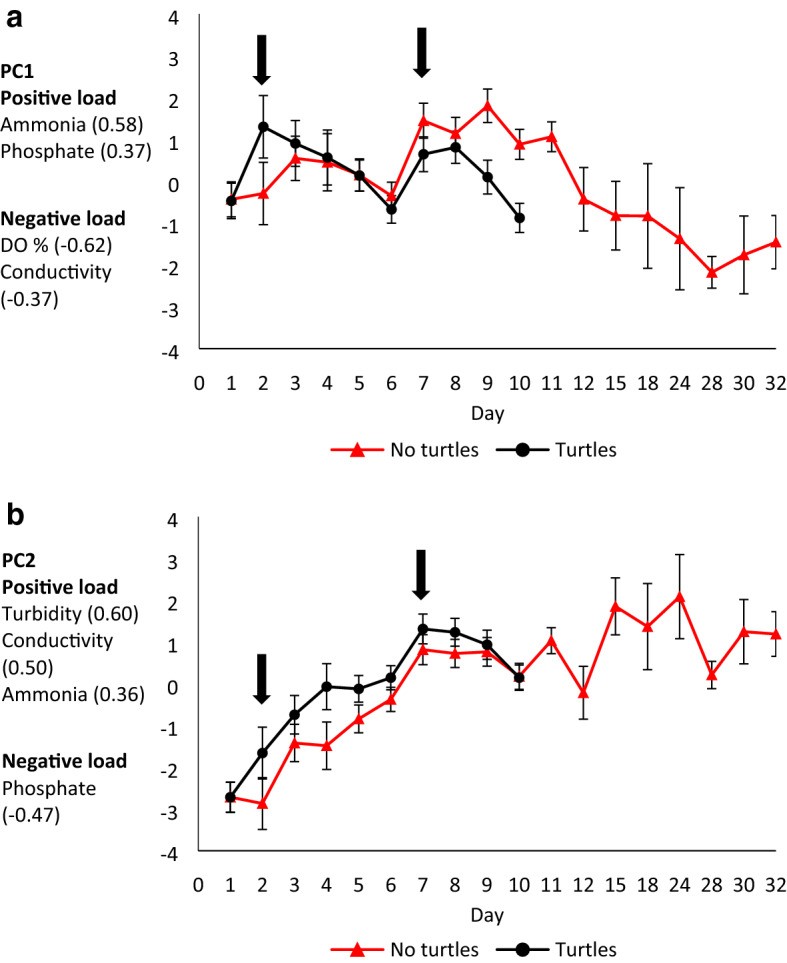
Estimates (mean ± SE) of PC1 (**a**) and PC2 (**b**), representing daily water quality measurements in mesocosms with turtles (black lines, dots) or without turtles (red lines, triangles). Arrows indicate the first day of data collection after each of the two carp carcasses were introduced. Next to the y-axis, the eigenvector of each water quality variable is reported in parentheses. We did not track water quality after day 10 in turtle mesocosms because carp carcasses were completely eaten, thus further changes would not be caused by their decomposition.

After the introduction of the first carp, PC1 increased more in turtle mesocosms, but returned to normal faster than in controls (Fig. 3a). After the second carp introduction, PC1 increased in both treatments, but again returned to pre-fish kill levels more quickly in turtle mesocosms (Fig. 3a). In contrast to PC1, PC2 increased after the introduction of carp in both treatments, and was higher in turtle mesocosms until the day before the carp were completely consumed (Fig. 3b). However, turbidity was the only parameter loading onto PC2 that was independently affected (positively) by the presence of turtles (interacting with day, F_9,102_ = 2.81, *p* = 0.006; Supplementary Table S8; Supplementary Fig. S2a).

Presence of turtles affected dissolved oxygen (interacting with day, F_9,102_ = 6.32, *p* < 0.001; Supplementary Table S8; Supplementary Fig. S2b), ammonia concentration (interacting with day, F_9,102_ = 3.97, *p* < 0.001; Supplementary Table S8; Supplementary Fig. S2c), as well as turbidity. There was no difference in conductivity and phosphate concentration between mesocosms with or without turtles (Supplementary Table S8; Supplementary Fig. S2d-2e).

## Discussion

Freshwater turtles provide an important ecosystem service as scavengers, so their global decline has cascading ramifications for ecosystems. All three species of freshwater turtle living in the Murray–Darling Basin scavenge^21–23^. Our experiments show that turtle scavenging mitigates the effect of animal decomposition on water quality, particularly during fish kills, and this ecological role diminishes as turtle abundance decreases.

Our field experiment shows that freshwater turtles are important scavengers in the Murray–Darling Basin, and that the more turtles present at a site, the greater the rate of carrion removal. This is the first time the scavenging role of freshwater turtles has been assessed experimentally. The presence of turtles significantly affects water chemistry and invertebrate biodiversity (e.g. red-eared sliders *Trachemys scripta elegans*^24^) but we show that, without turtles, carrion removal would likely be severely diminished. Crayfish, prawns, and shrimp are also scavengers in our system, but they were unable to replace turtle scavenging in our field experiment (Fig. 1). No other vertebrate scavenger was observed approaching the carp carcasses, nor were they caught in our traps, which are baited with offal, indicating that turtles were the only major scavengers in our experiment. However, we acknowledge that the scavenging role of other vertebrates, such as piscivorous fish, has not been studied in this system and cannot be ruled out. Regardless, water-breathing scavengers cannot replace the impact of turtles after a fish kill; they must compete for depleted oxygen with bacterial decomposers and increasing toxic biological nitrogen levels. Instead, they die of asphyxiation.

There is a clear link between water quality regulation and scavenging by *E. macquarii* (Fig. 3). The faster initial change in ammonia and DO in turtle mesocosms happened possibly because turtles dismembered carcasses and released flesh particles into the water column, where they were more accessible to bacteria. However, both ammonia and DO returned to initial conditions more quickly in turtle mesocosms compared to controls, where fish carcasses persisted for longer (Fig. 3, Supplementary Fig. S2b-2c). Both ammonia and DO are key metrics of water quality. Aquatic animals need DO for aerobic respiration^25^, so faster recovery of DO in turtle mesocosms means that turtle scavenging is likely to prevent aquatic animal mortality due to asphyxiation, which can occur during fish kills^5^. Turtles themselves would not be affected by a drop in DO because they breathe air. Ammonia concentration increases as a by-product of decomposition during fish kills^5^. Ammonia is toxic to animals^26^, and is an important nutrient for cyanobacterial blooms^27^. Thus, the faster decrease in ammonia concentration caused by turtle scavenging is likely to help prevent further aquatic animal mortality and reduce the extent of cyanobacterial blooms after a fish kill, thereby helping humans avoid resulting illnesses^6,8^.

We observed a steady rise in turbidity during the mesocosm experiment, particularly in the turtle mesocosms. During fish kills in nature, turbidity also increases^4,5^. It is likely that turtle dismembering of fish carcasses, and their metabolic wastes, increased turbidity compared to controls. In the controls, PC2 (primarily turbidity) continued to rise over time and peaked 24 days after the first carp was introduced (Fig. 3b). Notably, of the variables loaded on PC2, phosphate is the most important indicator of poor water quality as a nutrient for cyanobacteria^28^, but its concentrations did not increase as carp decomposed in either treatment (Supplementary Table S8; Supplementary Fig. S2e). Thus, phosphate concentrations did not reflect changes in water quality in this experiment, regardless of turtle scavenging.

Turtles have drastically declined in the Murray–Darling Basin^18,19^. Up to 100,000 tonnes of turtles were historically present^14^, but in some locations they are now so rare that they are not captured^19^. As turtle populations continue declining, we predict that water quality will worsen, with implications for both the ecosystem and humans. The Murray–Darling Basin supports 40% of Australia's agricultural production, and is the main source of water for more than 2.8 million people^29^. The possible release of CyHV-3 virus as a biocontrol for invasive carp is likely to produce massive carp mortality^30^, especially in areas of high carp density (e.g. the Lachlan river, ~ 3,144 kg carp/ha^31^). A sudden increase in carp carcasses where turtles are now scarce will severely affect the ecosystem due to the absence of scavengers removing them quickly^7^.

We conclude that the historically great abundance of turtles was critical for removing carrion rapidly before it decomposed, maintaining healthy freshwater ecosystems and hastening water quality recovery during fish kills. Any fish kill occurring in areas currently lacking abundant turtles is likely to have the worst outcomes. Thus, scavenging by turtles is a vital biological and economic freshwater ecosystem service. Worldwide, many species of freshwater turtle scavenge and are going extinct^16^*.* Therefore, we suggest turtle conservation and population recovery be prioritised and improved, both globally and in Australia, to restore the effects of their ecological role. In the Murray–Darling Basin, the control of introduced European foxes (*Vulpes vulpes*), measures to reduce turtle mortality on roads, and headstarting hatchlings are important conservation actions necessary to reverse the current decline^32^. As the basin has been significantly altered during the last century, these actions will need to be implemented alongside environmental management efforts that will support the return of turtle populations to their historical numbers, such as turtle-friendly flow regimes and restoration of aquatic vegetation.

## Methods

### Field experiment

#### Estimation of turtle catch per unit effort

We conducted our field experiment in February–April 2018 at two wetland complexes near Murray Bridge, South Australia, selecting two study sites at each complex (Supplementary Fig. S3). At each site, we estimated turtle population density using catch-per-unit-effort (CPUE; Supplementary Table S1). We conducted three 3-day rounds of turtle trapping using a combination of fyke and cathedral traps, baited with offal. Up to eight traps were deployed at a time. We calculated turtle CPUE by dividing the total number of turtles caught (regardless of species) by the total trap-hours. The number of trap-hours was similar across all four sites (average 1685 ± 7.6 SE total trap-hours).

#### Carp carcass decomposition

After the first and the second trapping rounds, we deployed whole carp carcasses at each site to measure carp decomposition rates depending on turtle accessibility. We placed each carp in a pre-weighed plastic box (340 × 230 × 120 mm), securing it with cable ties. Carp were made non-accessible to turtles in half of the deployments by covering the plastic boxes with 25 × 25 mm mesh (Supplementary Fig. S4). The mesh prevented turtle access to the carp, but was large enough to allow scavenging by crayfish (*Cherax destructor*) and other freshwater invertebrates. We tied each box to a brick and submerged the boxes around the four study sites ≥ 30 m away from each other, sunk at an average depth of 436 mm (± 13 SE). We used a total of 38 accessible and 40 non-accessible carp, split between our four study sites over two rounds (Supplementary Table S9). Every day, starting from day 2, the box and carp were weighed together with a digital scale. In all measurements, we calculated the wet mass of the carp by subtracting the box weight from the total weight. Carp carcasses were left in the wetlands for up to 10 days, or until they were fully consumed. All work was performed in accordance with DEWNR Permit M26663-1, PIRSA permits MP0085 and ME9902980, and The University of Sydney Animal Ethics Committee approval (project number 2017/1208), observing all relevant guidelines and regulations.

#### Statistical analysis

We analysed our data using RStudio 1.1.456^33^ (packages: “lme4” 1.1-21^34^, “MuMIn” 1.42.1^35^). To assess whether turtles were important scavengers of our carcasses, we computed a linear mixed model testing whether turtle CPUE and carp access (yes/no) affected the rate of mass loss of the carp carcasses. The turtle CPUE values used were the average CPUE in the trapping round before and after each carp was deployed. We used the rate of mass loss per day as a dependent variable. We included the carp mass before deployment as an independent variable to account for initial mass variation, and we included study site as a random variable. We log-transformed all data before analysis. We assessed model fit by examining predicted versus residual and Q–Q plots, and testing the normality of residuals.

### Mesocosm experiment

#### Turtle trapping and experiment procedure

We caught 20 adult male *E. macquarii* with fyke nets baited with offal at Hawkesview Lagoon, Albury, NSW, in November 2018. The *E. macquarii* captured at this site belong to the same genetic population as the *E. macquarii* trapped in South Australia^36^, therefore we expect behaviours to be similar between the two populations. We focussed on *E. macquarii* as this is the most common species in the Murray–Darling Basin, and fish carrion is an important part of its diet^19,37^. The turtles were transported by car to the Experimental Wetlands facility at Western Sydney University, in Richmond, NSW (Supplementary Fig. S5). This facility is comprised of 10 circular mesocosms (0.42 m depth × 2.1 m diameter) filled with 1,450 L of tap water. Each is an independent flow-through system where the water flow is regulated, and was maintained at 1998.6 ml/min (± 149.5 SE) throughout the experiment. Each mesocosm had two cement blocks for the turtles to bask on, and two plastic tunnels for shelter. The experiment was conducted for 40 days, therefore it is a short-term study (Supplementary Fig. S6). Upon arrival at the facility, we placed four adult male *E. macquarii* turtles in each of five random mesocosms, which means the experimental replication was 5. The remaining five mesocosms were controls and had no turtles. The four turtles comprised an average 5,376.6 g total biomass per pond, each being 3.46 m^2^. This would result in a biomass of 11,560 individuals/ha or 15,537 kg/ha on average. *Kinosternon integrum* has been estimated reaching densities of 20,000 individuals/ha in Sonora, Mexico, while *Podocnemis vogli* may reach 10,300 individuals/ha or 15,450 kg/ha in Venezuela, likely in temporary aggregations^38^. *Emydura macquarii* tend to congregate around food sources, therefore we considered four turtles per carp carrion as a realistic density. After 7 days of acclimation, we introduced one carp carcass to all mesocosms, and a second 6 days later. We used one ~ 1 kg carp at a time to simulate a density close to 3,144 kg carp/ha^31^. The turtles had continual access to the carp, which was their main food source throughout the experiment. The day all carp carcasses were fully eaten in all turtle mesocosms, we removed turtles from their mesocosms and released them at the point of capture. On the same day (day 10), we ended the data collection in their mesocosms, because any further change in water quality here would not have been related to carp decomposition. We continued the daily water quality measurements in the five control mesocosms until all carp were fully decomposed (day 32). This experimental design allowed us to collect water quality data without the need to add turtle food to the mesocosms, which would have biased our measurements once carp were removed from the turtle mesocosms.

We measured water temperature, dissolved oxygen, conductivity, turbidity, phosphate, and ammonia concentration in all mesocosms every morning from the day before the first carp introduction (see Supplementary Materials for equipment used). We also photographed the carcasses daily to estimate their decomposition rate based on a scale (Supplementary Table S10) designed after the decomposition stages described by Benninger et al.^39^ Due to the short transit time of fish matter in *E. macquarii*’s gut^37^, the effects of the turtles’ metabolic wastes on water quality are included in our experiment for carp 1. All work was performed in accordance with OEH Permit SL100401, DPI permit P09/0070-3.0, and Western Sydney University Animal Ethics Committee Animal Research Authority approval A12390, observing all relevant guidelines and regulations.

#### Statistical analysis

To assess whether the presence of turtles affected the decomposition of carp we computed a mixed linear model using the repeated measures PROC MIXED procedure using SAS (3.8 University Edition, SAS Institute Inc., Cary, NC, USA). For this model, days to total decomposition/removal was a dependent variable, turtle presence/absence was a fixed effect, and carp number (first or second) was a repeated fixed effect.

We used DO, conductivity, turbidity, phosphate, and ammonia to carry out a principal component analysis (PCA) using PROC PRINCOMP. We conducted a PCA because the parameters are a multivariate response and have potential to covary with each other, which would not be detected in univariate analyses. We considered a parameter loaded onto a PC when the absolute value of its eigenvector was > 0.300. If the same parameter loaded onto more than one PC, we considered it only on the PC where its eigenvector had a higher absolute value.

To test the effect of turtles scavenging on water quality, we computed general linear mixed models (GLMMs), using PCs as response variables, in PROC MIXED. We used a PC as response variable if its eigenvalue was greater than one (Kaiser criterion^40^). For each of these GLMMs, we included turtle presence (yes/no) and day number after the first carp introduction as fixed effects, water temperature and flow as covariates, and mesocosm ID as a random effect. We computed a model with full interactions first, and then, in absence of four- or three-way interactions, simplified the model to focus on main effects.

Finally, to test the effect of turtle scavenging on each water quality parameter, a GLMM was computed for each (logged) parameter that loaded onto a PC with eigenvalue > 1, i.e. dissolved oxygen, ammonia, turbidity, conductivity, phosphate. For these GLMMs, turtle presence and day number were fixed effects, water temperature and flow were covariates, and mesocosm ID was a random effect.

## Supplementary information

Supplementary Inormation.

## Data Availability

Datasets generated during the current study are available from the corresponding author on reasonable request.
