# Supplementary material for: Scavenging by threatened turtles regulates freshwater ecosystem health during fish kills
Source: Sci Rep. 2020 Sep 17;10:14383. doi: 10.1038/s41598-020-71544-3 (PMC7499268; doi:10.1038/s41598-020-71544-3)
Supplement: Supplementary file 1 — Supplementary Inormation. [file 41598_2020_71544_MOESM1_ESM.pdf]

Supplementary Materials for:

Scavenging by threatened turtles regulates freshwater ecosystem health during fish kills

Claudia Santori [A], Ricky-John Spencer [B]*, Michael B. Thompson [A], Camilla M. Whittington [A], Thomas H. Burd [A], Samantha B. Currie [B], Timothy J. Finter [B], James U. Van Dyke [C]

[A] School of Life and Environmental Sciences, The University of Sydney, Camperdown, NSW 2006, Australia;

[B] School of Science, Hawkesbury Institute for the Environment, Western Sydney University, Richmond, NSW 2753, Australia;

[C] School of Molecular Sciences, La Trobe University, Wodonga, VIC 3689, Australia.

*Correspondence to: r.spencer@westernsydney.edu.au

Supplementary methods

1.0 Field experiment

To prevent other potential scavengers (e.g. rakali *Hydromys chrysogaster*, flies, birds) from accessing the carp carcasses, we attached a brick to them, so that they rested on the bed of the wetland. A float was attached to each box to make it easier to find the carcass later and to observe its location from a distance. To monitor bird and rakali activity during the carp deployment, observations of 15 min each were conducted at each study site three times between 8:20 AM and 19:00 PM, at a location where the floats of at least six carp boxes were visible through binoculars. We did not observe any rakali at all, nor any water bird showed any attraction or dived in the proximity of the carcasses. On day 2, the carcasses were stabbed 2-3 times with a knife to prevent floating. Because of strong wind on day 3 of the second round, we were unable to weigh 16 carp carcasses, and two carp boxes in Paiwalla B floated to the bank. We returned these two carp boxes to the water, sank them, and restarted the weight monitoring from that day forward.

1.1 Statistical analysis of field experiment

In the linear mixed model to test whether turtle CPUE and carp access (yes/no) affected the rate of mass loss of the carp carcasses, we used the average CPUE in the trapping round before and after each carp was deployed. For example, if carp #2 was deployed during the first carp deployment round, the CPUE assigned to this carp was the average between the CPUE of the first and second turtle trapping rounds at the site where the carp was deployed.

2.0 Mesocosm experiment

We split the turtles evenly according to weight so that each mesocosm had an average total turtle weight of 5.38 kg (± 0.02 SE). We measured temperature and dissolved oxygen (%) with a YSI ProODO digital professional series (John Morris scientific), conductivity (µS) with an Aqua-CP Conductivity/pH meter (TPS), turbidity (NTU) with a 2100P Turbidimeter 46500-00 (HACH), phosphate PO_4^{3-} (mg/L) with a DREL/2400 spectrophotometer (HACH) and PhosVer 3 (ascorbic acid) powder pillows (PERMACHEM), and total ammonia NH_3/NH_4^+ concentration (ppm) with an API® aquarium test kit.

Supplementary figures and tables

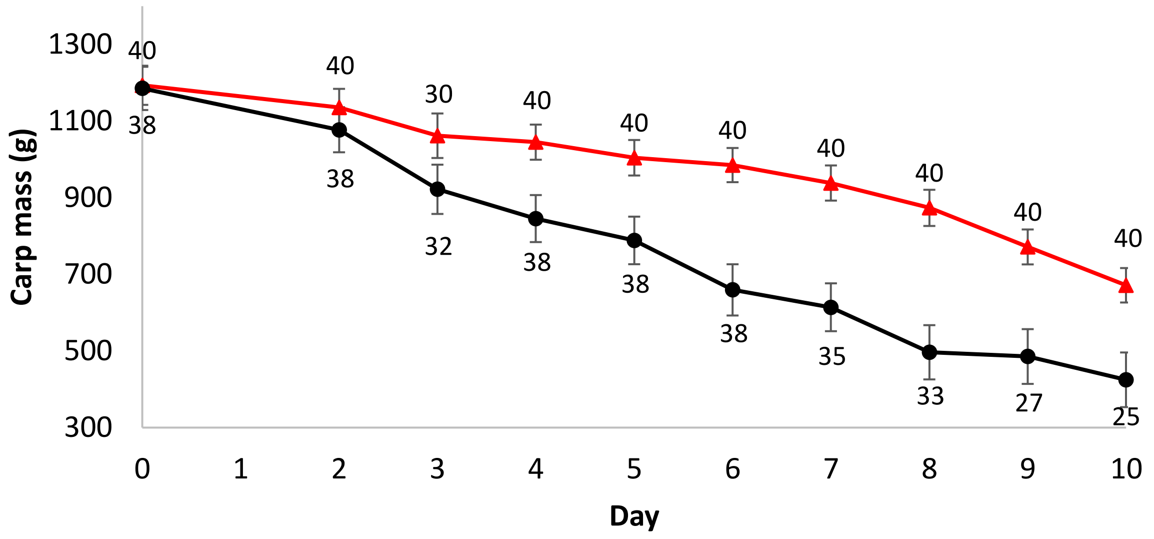

Figure S1. Average mass of accessible carp carcasses (black dots) decreased more over time, and more quickly, compared to the average mass of non-accessible carp carcasses (red triangles). The number above each dot represents the number of carcasses still present and not completely removed. Carp were removed from the graph when they were completely eaten (only bones remaining) or they fully disappeared from the box. N = 10 non-accessible carp and N = 6 accessible carp were not sampled on day 3 due to adverse weather.

a

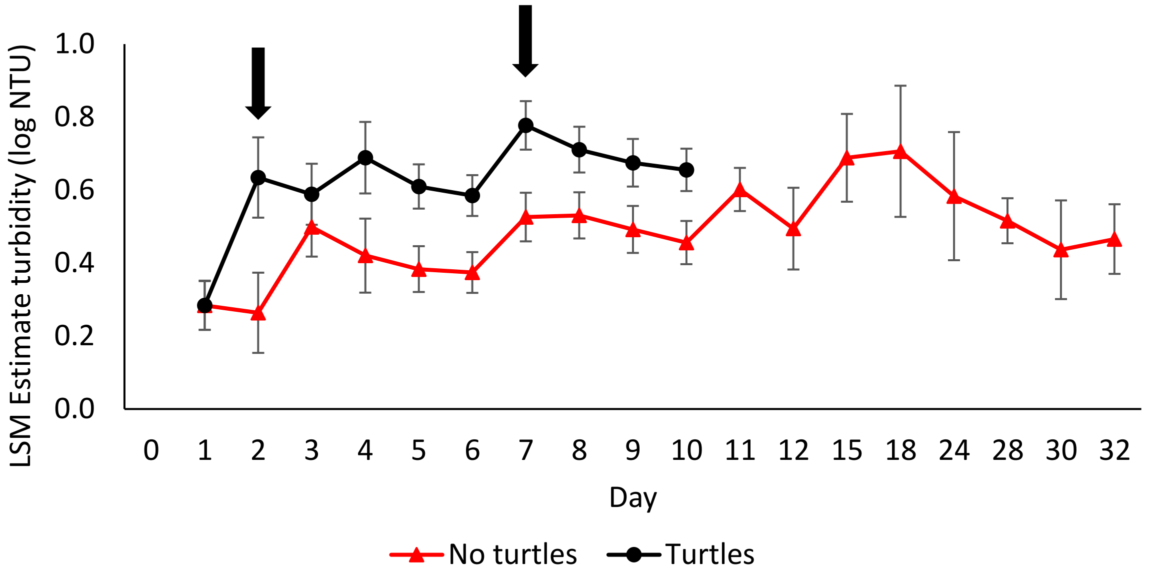
1.0
0.8
0.6
0.4
0.2
0.0
LSM Estimate turbidity (log NTU)
0 1 2 3 4 5 6 7 8 9 10 11 12 15 18 24 28 30 32
Day
No turtles
Turtles

b

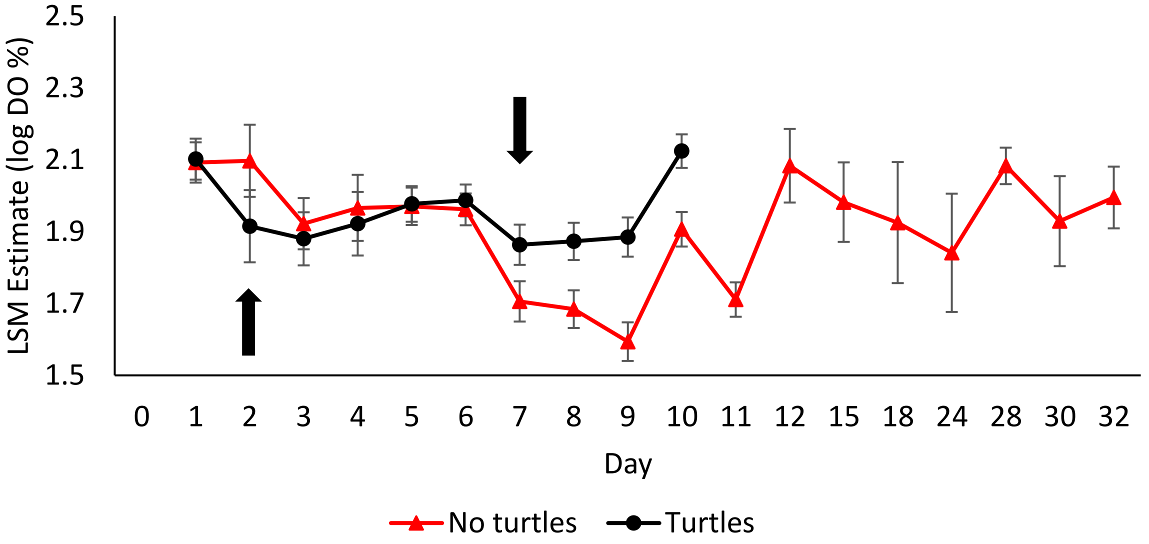
2.5
2.3
2.1
1.9
1.7
1.5
LSM Estimate (log DO %)
0 1 2 3 4 5 6 7 8 9 10 11 12 15 18 24 28 30 32
Day
No turtles
Turtles

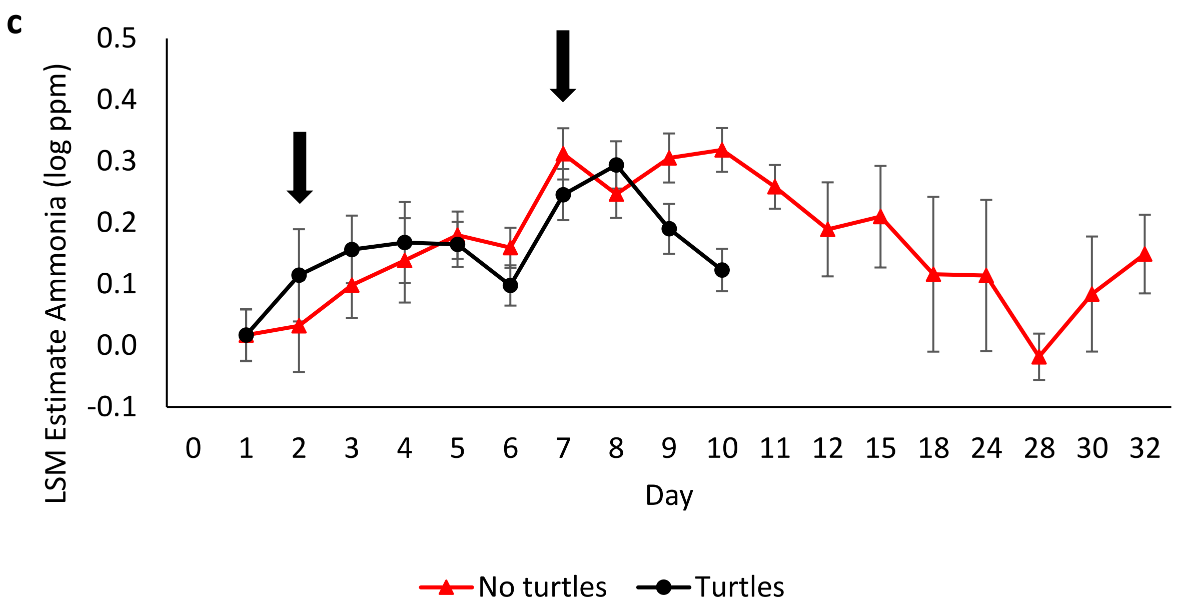
c
0.5
0.4
0.3
0.2
0.1
0.0
-0.1
LSM Estimate Ammonia (log ppm)
0 1 2 3 4 5 6 7 8 9 10 11 12 15 18 24 28 30 32
Day
No turtles
Turtles

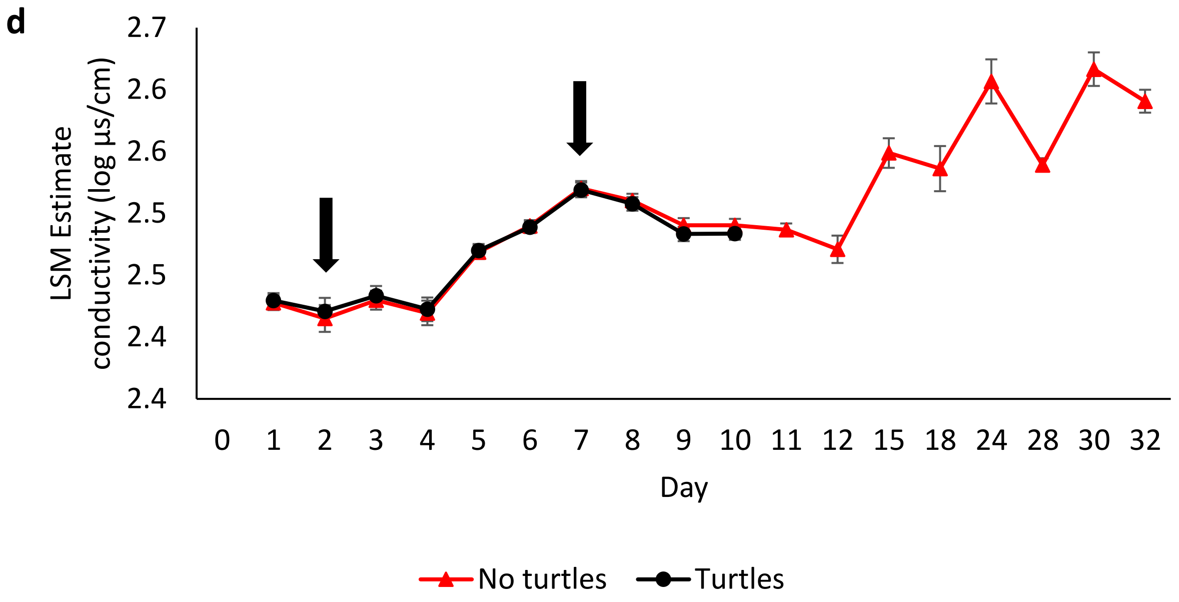
d
2.7
2.6
2.6
2.5
2.5
2.4
2.4
LSM Estimate conductivity (log µs/cm)
0 1 2 3 4 5 6 7 8 9 10 11 12 15 18 24 28 30 32
Day
No turtles
Turtles

e

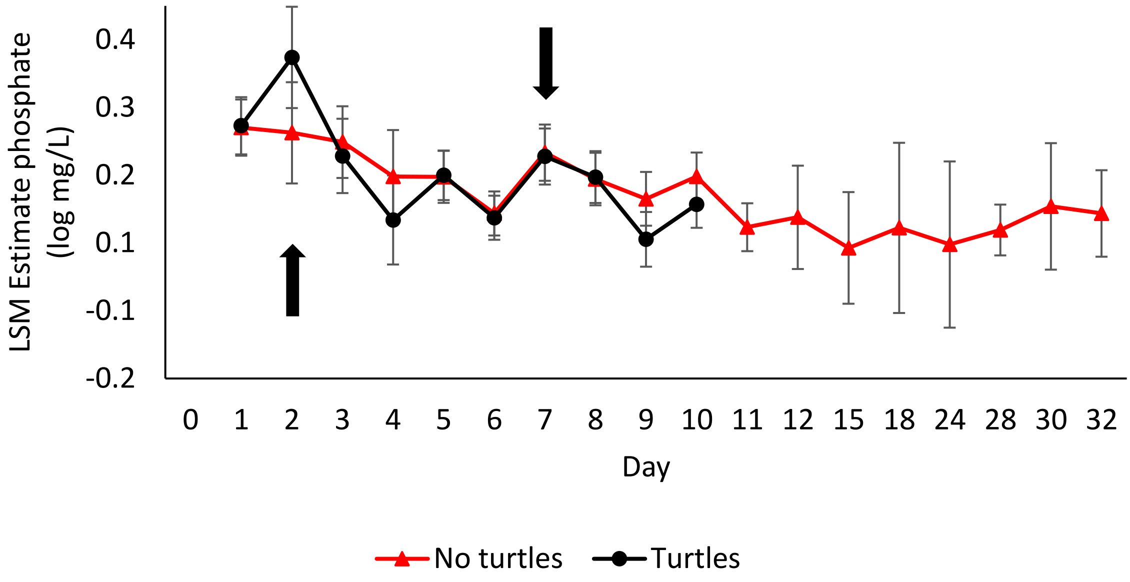

Figure S2. Least square means (LSM) estimates of each of the water parameters measurements (mean ± SE), calculated for each GLMM (Table S10). The water parameters represented in each sub-Figure are a) = turbidity (log NTU); b) = dissolved oxygen (log %); c) = ammonia (log ppm); d) = conductivity (log µs/cm); e) = phosphate (log mg/L). Arrows indicate the first day of data collection after the carp carcasses were introduced to the 10 mesocosms.

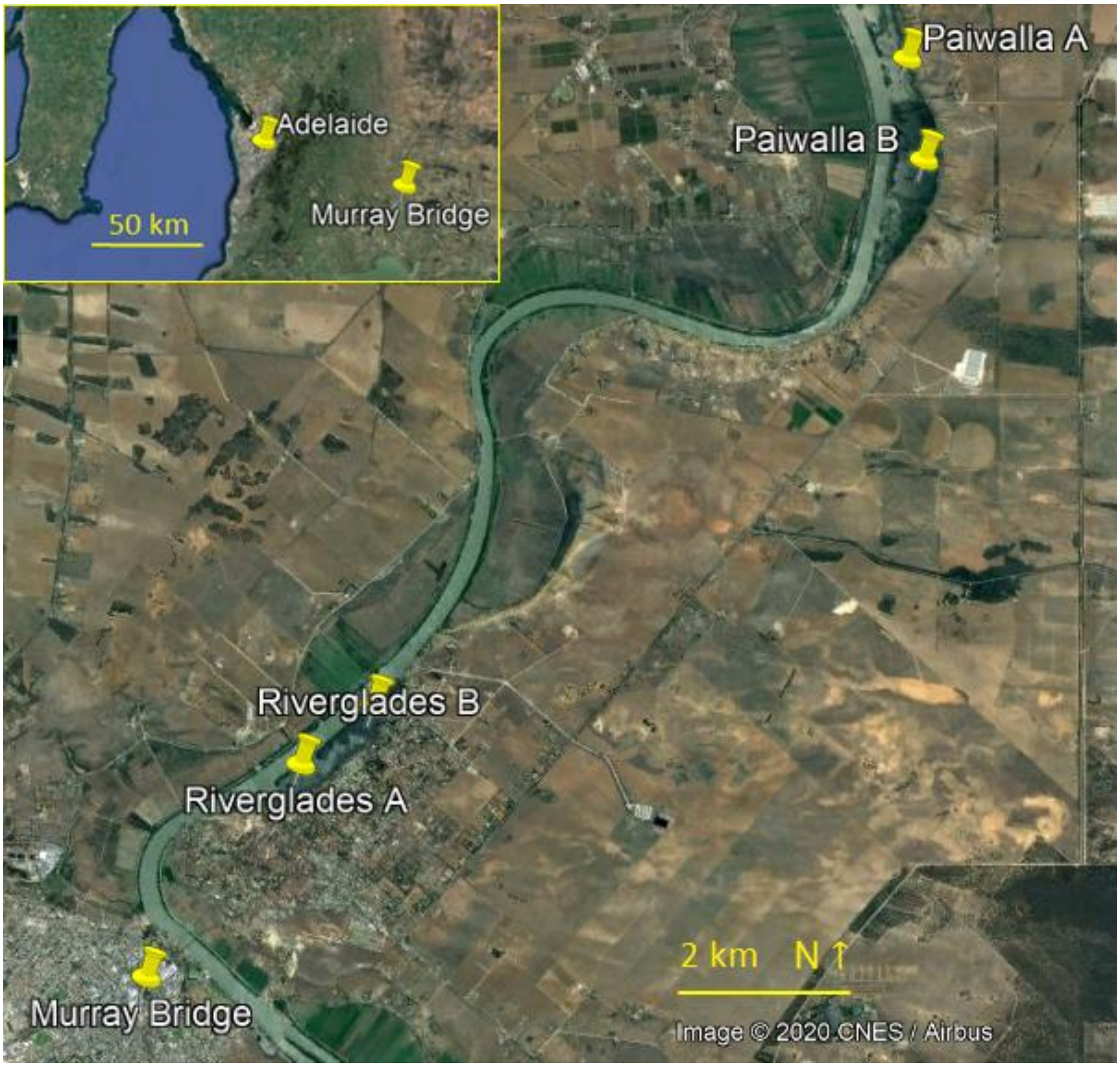

Figure S3. Map of field sites, located in proximity of the Rural City of Murray Bridge, South Australia. Riverglades (35° 5' 49.51" S, 139° 17' 58.06" E) and Paiwalla (35° 2' 13.17" S, 139° 22' 20.77" E). Images obtained from Google Earth Pro (version 7.3.3). Top image: Landsat/Copernicus, eye altitude 301.4 km, SIO, NOAA, U.S. Navy NGA, GEBCO. Bottom image: CNES/Airbus 2020, eye altitude 17.5 km, imagery date 11/17/2018. http://www.earth.google.com. Both accessed 27/07/2020.

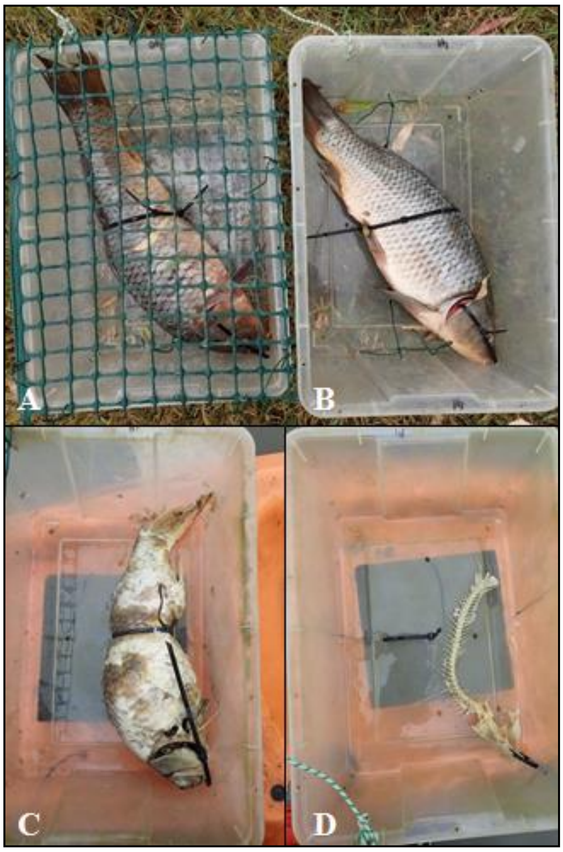

Figure S4. An example of carp non-accessible and accessible to turtles (A, B) right before deployment, and an example of non-accessible (after mesh removal) and accessible carp (C, D) after 7 days in the water.

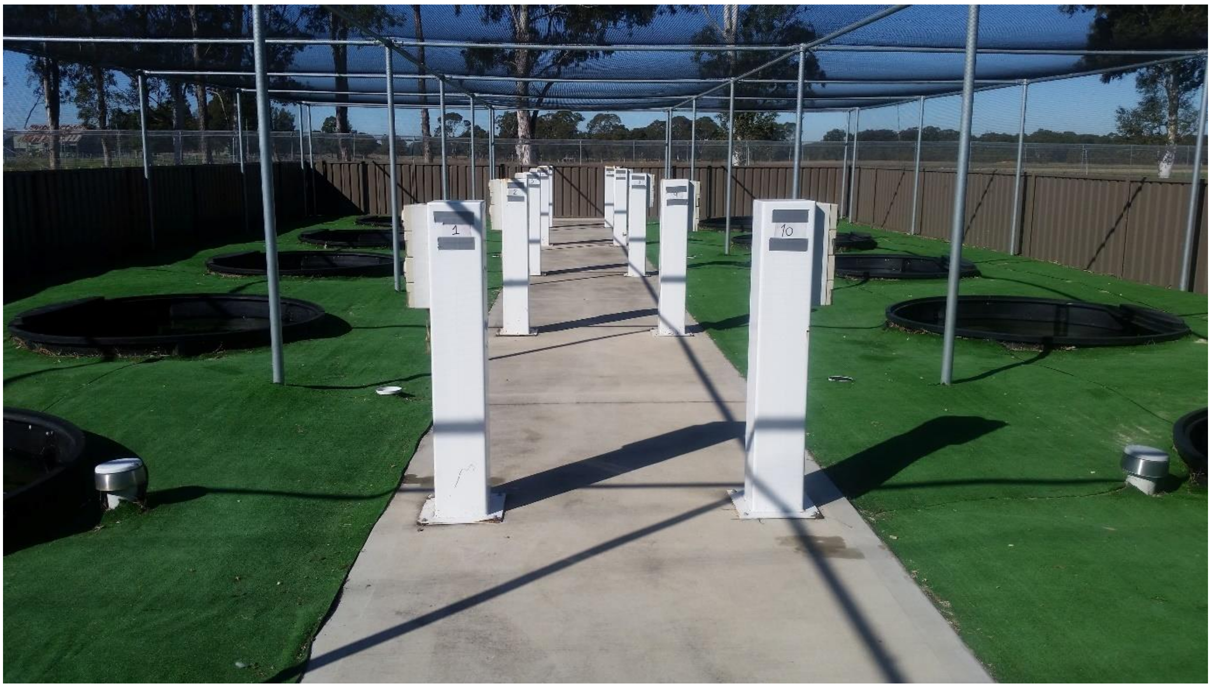

Figure S5. Experimental Wetlands facility, showing predator- and bird-proof fencing.

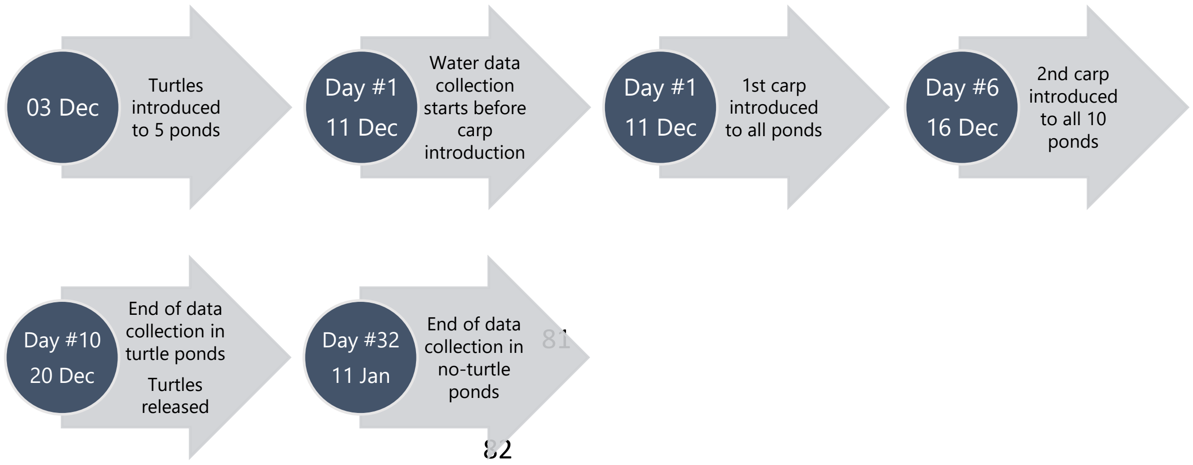

Figure S6. Timeline of mesocosms experiment. Water quality data were always collected in the morning, while the carp carcasses were introduced in the afternoon. Ponds = mesocosms.

Table S1. Numbers of turtles caught for each species at each site over the three trapping rounds, and average combined CPUE (turtles/trap/hour) for rounds 1-2 and 2-3 used in the model. A-sites are open to the Murray River, and B-sites were closed to the river.

	C. expansa	*C. longicollis*	*E. macquarii*	Average CPUE rounds 1-2	Average CPUE rounds 2-3
Riverglades A	12	22	39	0.04	0.05
Riverglades B	1	16	5	0.02	0.01
Paiwalla A	3	6	39	0.03	0.02
Paiwalla B	0	7	6	0.01	0.00

Table S2. Type 3 tests of fixed effects of turtle presence/absence in the mesocosms (“turtles”) and carp number (first or second, “carp n”) on the number of days to total decomposition.

Effect	Num DF	Den DF	F Value	Pr > F
Turtles	1	8	482.65	<0.001
Carp n	1	8	19.53	0.002
Turtles * Carp n	1	8	0.28	0.610

Table S3. Eigenvalues of the principal components (PC) correlation matrix of the mesocosm experiment. Water quality variables input in the PCA: DO, ammonia, conductivity, turbidity, and phosphate.

PC	Eigenvalue	Difference	Proportion	Cumulative
1	1.91	0.52	0.38	0.38
2	1.39	0.60	0.28	0.66
3	0.79	0.18	0.16	0.82
4	0.61	0.32	0.12	0.94
5	0.29	0.06	1.00	

Table S4. Type 3 tests of fixed effects of turtle presence/absence in the mesocosms ("turtles"), day after carp introduction ("day"), water temperature ("temp") and water flow rate ("flow") on PC1, allowing four-way interactions. All explanatory variables apart from temperature and flow were logged prior to the analysis.

Effect	Num DF	Den DF	F Value	Pr > F
Turtles	1	23	7.41	0.01
Day	16	23	1.68	0.12
Day * Turtles	6	23	1.88	0.13
Temp	1	23	0.15	0.70
Temp * Turtles	1	23	2.72	0.11
Temp * Day	16	23	1.73	0.11
Temp * Day * Turtles	8	23	1.71	0.15
Flow	1	23	0.26	0.61
Flow * Turtles	1	23	2.74	0.11
Flow * Day	16	23	1.71	0.12
Flow * Day * Turtles	8	23	1.92	0.11
Temp * Flow	1	23	0.19	0.67
Temp * Flow * Turtles	1	23	2.53	0.13
Temp * Flow * Day	17	23	1.87	0.08
Temp * Flow * Day * Turtle	8	23	1.91	0.11

Table S5. Type 3 tests of fixed effects of turtle presence/absence in the mesocosms (“turtles”), day after carp introduction (“day”), water temperature (“temp”) and water flow rate (“flow”) on PC2, allowing four-way interactions. All explanatory variables apart from temperature and flow were logged prior to the analysis.

Effect	Num DF	Den DF	F Value	Pr > F
Turtles	1	23	0.02	0.89
Day	16	23	1.81	0.10
Day * Turtles	6	23	1.80	0.14
Temp	1	23	0.49	0.49
Temp * Turtles	1	23	0.05	0.82
Temp * Day	16	23	1.35	0.25
Temp * Day * Turtles	8	23	1.75	0.14
Flow	1	23	0.64	0.43
Flow * Turtles	1	23	0.01	0.93
Flow * Day	16	23	1.51	0.18
Flow * Day * Turtles	8	23	1.45	0.23
Temp * Flow	1	23	1.15	0.30
Temp * Flow * Turtles	1	23	0.01	0.92
Temp * Flow * Day	17	23	1.61	0.14
Temp * Flow * Day * Turtle	8	23	1.45	0.23

Table S6. Type 3 tests of fixed effects of turtle presence/absence in the mesocosms ("turtles"), day after carp introduction ("day"), water temperature ("temp") and water flow rate ("flow") on PC1, reduced to two-way interactions (between "day" and "turtles", and between "temperature" and "flow"). All explanatory variables apart from temperature and flow were logged prior to the analysis.

Effect	Num DF	Den DF	F Value	Pr > F
Turtles	1	102	1.61	0.207
Day	17	102	11.99	<0.001
Day * Turtles	9	102	5.27	<0.001
Temp	1	102	0.36	0.548
Flow	1	102	0.01	0.907
Temp * Flow	1	102	0.00	0.989

Table S7. Type 3 tests of fixed effects of turtle presence/absence in the mesocosms ("turtles"), day after carp introduction ("day"), water temperature ("temp") and water flow rate ("flow") on PC2, reduced to two-way interactions (between "day" and "turtles", and between "temperature" and "flow"). All explanatory variables apart from temperature and flow were logged prior to the analysis.

Effect	Num DF	Den DF	F Value	Pr > F
Turtles	1	102	5.45	0.022
Day	17	102	22.21	<0.001
Day * Turtles	9	102	1.84	0.070
Temp	1	102	0.00	0.959
Flow	1	102	3.90	0.051
Temp * Flow	1	102	4.07	0.046

Table S8. Type 3 tests of fixed effects of turtle presence/absence in the mesocosms ("turtles"), day after carp introduction ("day"), water temperature ("temp") and water flow rate ("flow") on the individual water quality parameters loading onto PC1 and/or PC2, allowing two-way interactions (between "day * turtles" and "temperature * flow"). All explanatory variables apart from temperature and flow were logged prior to the analysis.

	Effect	Num DF	Den DF	F Value	Pr > F
	Turtles	1	102	4.28	0.04
	Day	17	102	12.71	<0.001
**DO %**	Day * Turtles	9	102	6.32	<0.001
	Temp	1	102	0.81	0.37
	Flow	1	102	0.64	0.43
	Temp * Flow	1	102	0.32	0.57
	Turtles	1	102	1.26	0.26
	Day	17	102	11.83	<0.001
**Ammonia**	Day * Turtles	9	102	3.97	<0.001
	Temp	1	102	0.00	0.96
	Flow	1	102	2.24	0.14
	Temp * Flow	1	102	2.21	0.14
	Turtles	1	102	12.79	<0.001
	Day	17	102	8.44	<0.001
**Turbidity**	Day * Turtles	9	102	2.81	0.006

	Temp	1	102	0.01	0.92
	Flow	1	102	0.19	0.66
	Temp * Flow	1	102	0.13	0.72
	Turtles	1	102	0.03	0.87
	Day	17	102	57.85	<0.001
**Conductivity**	Day * Turtles	9	102	0.44	0.91
	Temp	1	102	2.47	0.12
	Flow	1	102	3.69	0.06
	Temp * Flow	1	102	3.62	0.06
	Turtles	1	102	0.15	0.70
	Day	17	102	4.76	<0.001
**Phosphate**	Day * Turtles	9	102	1.28	0.26
	Temp	1	102	0.81	0.37
	Flow	1	102	3.12	0.08
	Temp * Flow	1	102	3.31	0.07

Table S9. Count of carp carcasses deployed at each site and round.

	Site	Accessible	Not accessible	Total
First round				**38**
	Paiwalla A	1	5	6
	Paiwalla B	6	6	12
	Riverglades A	6	6	12
	Riverglades B	5	3	8
Second round				**40**
	Paiwalla A	9	5	14
	Paiwalla B	3	3	6
	Riverglades A	4	5	9
	Riverglades B	4	7	11
Total		**38**	**40**	

Table S10. Carp decomposition assessment chart based on Benninger *et al.* (2008) [39].

Percentage decomposed	Description	Example photograph
0%	Fully intact, no visible decomposition, floating	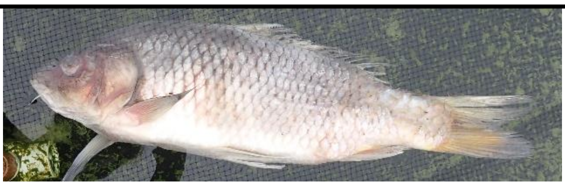
25%	Scales falling off, fins decomposing, discolouration, floating	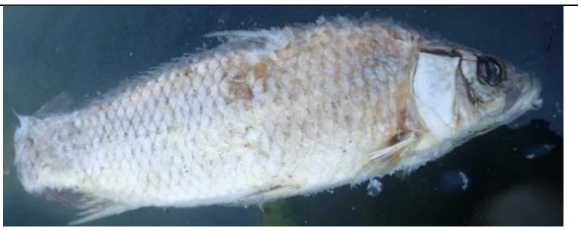
50%	Flesh decomposing, skeleton exposed, sunk	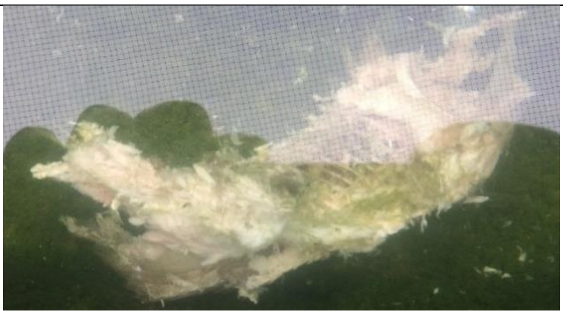
75%	Most of the flesh gone, skeleton and skin remaining, sunk	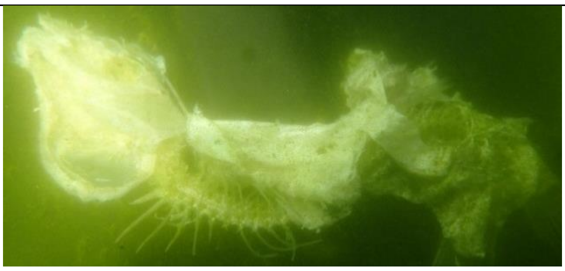
100%	Only skeleton remaining	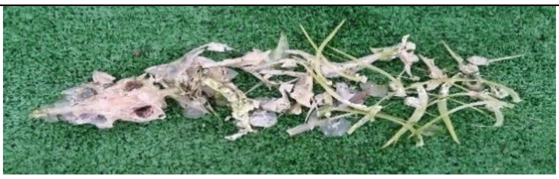
